# Diagnosis of brain death and consecutive donor management under combined circulatory support with ECMELLA therapy

**DOI:** 10.3389/fmed.2025.1569951

**Published:** 2025-03-20

**Authors:** Florian J. Raimann, Laurent M. Willems

**Affiliations:** ^1^Department of Anesthesiology, Intensive Care Medicine and Pain Therapy, University Hospital, Goethe University Frankfurt, Frankfurt, Germany; ^2^Department of Neurology and Epilepsy Center Frankfurt Rhine-Main, University Hospital, Goethe University Frankfurt, Frankfurt, Germany

**Keywords:** ECMO, impella, critical care, cardiac arrest, intensive care, organ donation

## Abstract

**Background:**

Managing brain death determination (BDD) in potential organ donors is a challenging aspect of modern intensive care medicine. In critically ill patients with implanted circulatory or left ventricular support devices, standard recommendations for BDD are often no longer applicable.

**Methods/results:**

The available recommendations and evidence for BDD and organ procuring under ECMELLA therapy—a combined circulatory support using a veno-arterial extracorporeal membrane oxygenation (vaECMO) and an invasive left ventricular support device (Impella^®^ CP)—are discussed based on a clinical case. To the authors’ knowledge, this is the first report of BDD under ECMELLA therapy.

**Conclusion:**

Although BDD in patients with multimodal invasive circulatory support, such as ECMELLA therapy, is demanding and time-intensive, it can still be performed safely and based on evidence. Given the continuing low numbers of organ donors, these insights may help to facilitate organ donation in patients with combined invasive mechanical circulatory support.

## Background

Brain death determination (BDD) is a central yet challenging aspect of modern interdisciplinary intensive care medicine. National and international guidelines strictly regulate BDD, providing clear instructions for the procedure and interpretation of findings in most cases (1). The use of extracorporeal support devices can sometimes make the diagnosis of BDD considerably more difficult or even impossible. Over the last few years, extracorporeal cardiopulmonary resuscitation (eCPR) and extracorporeal life support (ECLS) have been increasingly implemented in CPR and post-resuscitation care. Moreover, invasive short-term cardiac assist devices, such as micro-axial flow pumps (e.g., the Impella CP^®^), have been established to bridge patients with insufficient or critical cardiac function after the return of spontaneous circulation (ROSC). Both procedures significantly complicate BDD and donation after brain death (DBD) considerably and are often not adequately addressed in guidelines (2, 3).

**Figure 1:**
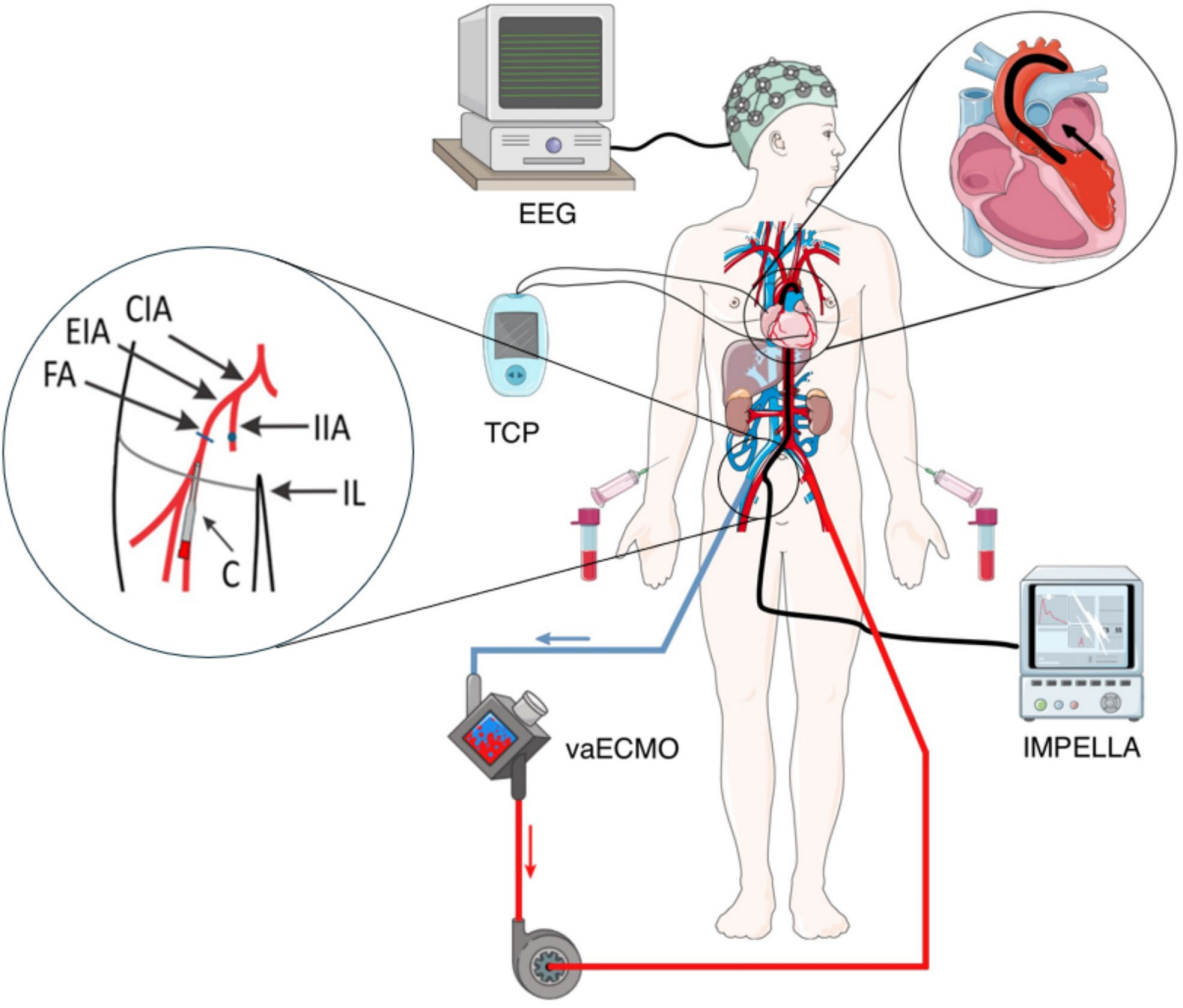
Schematic overview of brain death determination (BDD) in the described patient with multimodal cardiocirculatory support and transcutaneous pacer (TCP). CIA = common iliac artery, EIA = external iliac artery, FA = femoral artery, IIA = internal iliac artery, IL inguinal, and C = (arterial return) cannula [figure based on images from Servier Medical ART, licensed under CC BY 4.0, https://smart.servier.com].

This brief perspective illustrates and discusses the implications and potential pitfalls of a combined vaECMO (CardioHelp^®^; Getinge Deutschland GmbH, Rastatt, Germany) and Impella^®^ + SmartAssist (Abiomed Europe, Aachen, Germany) (ECMELLA) therapy on BDD and DBD based on the case of a 54-year-old man who developed low cardiac output after complex Tirone-David operation (aortocoronary bypass + aortic valve replacement). In addition to an intraoperative epicardial pacer, the patient consecutively received a vaECMO [ELSO Maastricht nomenclature: V23/55frivc-A17/15fldt (4)] under transient cardiopulmonary resuscitation (CPR) as well as an Impella^®^ CP due to ongoing cardiac failure (Figure 1). Due to inadequate clinical awakening and signs of brainstem dysfunction, a cranial CT was performed, revealing global hypoxic brain damage with generalized cerebral edema. The unfavorable prognosis and the patient’s family’s willingness for DBD were discussed with the legal representative, and BDD was initiated according to the national guidelines, as the concept of donation after cardiac death (DCD) has not been approved in Germany (1, 5). A schematic overview of the clinical and device instead of aparitive setting on intensive care unit is given in Figure 1.

## Results

### Implications of ECMELLA on brain death determination

The German guidelines specify a strictly regulated two-step process for BDD, comprising primarily the clinical confirmation of the loss of brain functions and secondary proof of irreversibility for donation after brain death (DBD) (1, 6). To avoid false high CO_2_ values due to a Harlequin effect under vaECMO samples for arterial blood gas analysis (BGA) were drawn from both brachial arteries following national and international recommendations. In the present case, the Harlequin effect was further enhanced by the orthogonal flow of the Impella^®^ device, and both Impella^®^ and vaECMO settings had to be adjusted in multiple small steps to facilitate bilateral CO_2_ values of 35 to 45 mmHg as basic requirements for conducting the apnea test. Once the initial paCO_2_ values were within the specified range, the vaECMO and Impella flow rates were carefully and gradually reduced. An increase in CO_2_ to >60 mmHg could only be achieved through a gradual reduction in O_2_ admixture and flow. Due to the increased risk of organ hypoxemia after more than 9 min, a minimal FiO_2_ had to be maintained during this procedure. Due to the high risk of clotting in the oxygenator during flow reduction, the flow could only be reduced carefully while on standby for manual operation. After adjusting the paCO_2_ to >60 mmHg in both BGAs obtained from the brachial artery lines, the apnea test was performed. Notably, the apnea test had to be discontinued several times due to cardio-circulatory insufficiency caused by hypercapnic acidosis. The presented approach was orientated on the “Guidance for the Diagnosis of Death using Neurological Criteria when the patient is supported with extracorporeal membrane oxygenation” (7). Furthermore, after pausing the epicardial pacemaker [heart rate: 90–100 beats*min^−1^, AV delay 140 ms, atrial pacing 10 V, ventricular pacing 12 V (transcutaneous pacer)], asystole was observed. Given this and the absence of blood pressure autoregulation under ECMELLA therapy [Impella CP: Flow Control P3, Purge Flow 16.5 mL*h^−1^, Purge Pressure 400–450 mmHg, Impella Flow 1.6 L*min^−1^; vaECMO: 3000RPM, 2.8 LPM, FiO2 0.9, SweepGas Flow 2.0 L*min^−1^], the assessment of a vegetative reaction to painful stimuli, such as an increase in heart rate or systolic blood pressure, was deemed unreliable. Consequently, observation of vegetative response to nociceptive stimuli was reduced to potential motor reactions, facial grimacing or increased sweating. Finally, the clinical loss of brain function was identified.

Under vaECMO therapy, and therefore also under ECMELLA, confirmation of the irreversibility of brain function loss was accessed by electroencephalography (EEG). Following national and international guidelines, proof of irreversibility through cerebral circulatory arrest was not permissible due to the low mean systolic pressure under mechanic circulatory support, as a mean arterial pressure of >90 mmHg is required as a fundamental prerequisite (1). Alternatively, confirmation of irreversibility by means of a second clinical protocol with an apnea test after >72 h was not considered feasible due to the patient’s instability and the abovementioned significantly complicated procedure. For these reasons, irreversibility was determined by EEG in the present case. Due to multiple sources of electrical interference and fields under ECMELLA, as well as the cardiac pacer and other electrical devices within the ICU environment, EEG sampling using Ag/AgCl-surface electrodes was not feasible for technical reasons. Consequently, platinum needle electrodes were inserted into the scalp according to the 10–20 system (8). To further reduce artifacts caused by cables and supply lines in the bed, the electrodes were routed to hang freely at the head end of the bed, ensuring the cables make contact with the bed or the patient, except at their tips. As a result of these measures, an artifact-free recording was achieved, producing an isoelectric EEG < 2 μV without evidence of brain activity thus confirming brain death (1).

### Implications of ECMELLA on organ donation management

For the perfusion of the abdominal organs, 8 L (800 mL/min application rate) of a Custodiol^®^ solution (Dr. Franz Köhler Chemie GmbH, Bensheim, Germany) was used. Based on the ECMELLA treatment, three different perfusion scenarios were discussed in advance between the anesthesiology team, cardio-technicians, and visceral surgeons.

1. Active perfusion via vaECMO over the draining cannula (see Figure 1 – return cannula)
2. Passive, gravity-following perfusion via the arterial return cannula (17 Fr) (see Figure 1 – return cannula)
3. Passive, gravity-following perfusion via the external iliac artery (see Figure 1 – blue dot)

Option 3, described in Table 1, was selected as the primary route to organ perfusion after interdisciplinary discussion as all team members are familiar with this procedure and it represents the lowest risk of complications. Option 2 was defined as the relapse option as the perfusion speed is higher here compared to option 3. During organ and vessel preparation, it was noticed that the return cannula, around which a ligature would have to be placed, extends too short into the abdomen from the intra-abdominal area.

**Table 1:** Dis−/advantages of perfusion procedures.

Option	Advantage	Disadvantage
1	Rapid application of the perfusion solution	Necessity to install a 3/8′-3/8’ LuerLock T-piece (see Figure 1) Short-term clamping of the connecting tube between the drainage cannula and oxygenator Insufficient lumen of the LuerLock
2	Rapid application of the perfusion solution	Need to install a 3/8′-1/4′ adapter (see Figure 1) Stopping the vaECMO and cutting the tube between the oxygenator and the return cannula Ligature around the drainage cannula at a length of 15 cm and deep seat in the area of the inguinal ligament
3	Parallel set-up of all materials required for perfusion. No ligature around the return cannula Perfusion procedure already familiar to all team members	Slower application of the perfusion solution

## Discussion

This complex scenario of brain death diagnostics and intraoperative management under ECMELLA therapy is rare. Due to its infrequency and the unique challenges posed by the therapy, physicians face significant difficulties. Not only is a high level of expertise in brain death diagnosis essential but also extensive knowledge in managing vaECMO and Impella is highly relevant. These challenges persist throughout the organ donation process. For instance, explantation surgeons must have a thorough knowledge of the perfusion cannula position and its resulting implications. In our view, an interdisciplinary preliminary discussion and dedicated planning of both BDD and explantation are crucial to avoiding complications. In the context of BDD under vaECMO therapy, the adjustment of guideline-compliant paCO2 partial pressures for the correct performance of the apnea test poses a particular challenge (9–11), as attention must be paid to the occurrence of Harlequin syndrome. The Harlequin effect is a condition that can occur with peripheral cannulation during veno-arterial extracorporeal membrane oxygenation (VA-ECMO). In cases of poor lung function coupled with residual cardiac contractility, deoxygenated blood enters the aortic arch, leading to reduced perfusion in the upper extremities as hypoxemic blood reaches the upper half of the body. This occurs due to antegrade (hypoxemic) blood flow, which flows against the retrograde blood flow generated by the ECMO. The IMPELLA placed over the aortic valve can enhance the Harlequin effect by artificially increasing the antegrade flow, even in the absence of residual myocardial contractility. Bilateral blood gas analyses on both upper extremities are necessary, requiring distinct knowledge of ECMO and IMPELLA operation. Another complicating factor is that physiological parameters continue to change under combined therapy with Impella^®^ CP. Among other things, the localization of the support devices as well as of their cannulation is crucial here (12). Although reviews on brain death diagnostics under vaECMO already exist (11, 13, 14), there are currently no guidelines or procedural instructions for managing brain death diagnosis under ECMELLA therapy. To our knowledge, the procedure presented here is the first description of brain death diagnostics under ECMELLA therapy. We hope that our approach, experiences, and thoughts will help others in improving BDD under ECLS with ECMO and IMPELLA devices.

## Data Availability

The original contributions presented in the study are included in the article/supplementary material, further inquiries can be directed to the corresponding author.

## References

[1] Angstwurm H Bartenstein PA Brandt S Clusmann H Emami P Förderreuther S. German guideline for brain death diagnosis (5th updated version) In: Official announcement of the FMA. Berlin, Germany: German Federal Medical Association (2022). 1–31.

[2] Sandroni C D'Arrigo S. Brain death is common after extracorporeal cardiopulmonary resuscitation (eCPR): an undesired outcome with potential benefits. Resuscitation. (2024) 200:110246. doi: 10.1016/j.resuscitation.2024.110246, PMID: 38768678

[3] Rajsic S Treml B Innerhofer N Eckhardt C Radovanovic Spurnic A Breitkopf R. Organ donation from patients receiving extracorporeal membrane oxygenation: a systematic review. J Cardiothorac Vasc Anesth. (2024) 38:1531–8. doi: 10.1053/j.jvca.2024.03.020, PMID: 38643059

[4] Broman LM Taccone FS Lorusso R Malfertheiner MV Pappalardo F Di Nardo M. The ELSO Maastricht treaty for ECLS nomenclature: abbreviations for cannulation configuration in extracorporeal life support—a position paper of the extracorporeal life support organization. Crit Care Med. (2019) 23. doi: 10.1186/s13054-019-2334-8, PMID: 30736845 PMC6367794

[5] Eden J Dutkowski P Schlegel A. Reply to: "how many liver grafts could be recovered after implementation of donation after cardiac death in Germany?". J Hepatol. (2023) 79:e120–1. doi: 10.1016/j.jhep.2023.05.014, PMID: 37245704

[6] Hoffmann O Salih F Masuhr F. Diagnosis of brain death in Germany-implementation of the guidelines of the German Medical association. Nervenarzt. (2023) 94:1129–38. doi: 10.1007/s00115-023-01520-5, PMID: 37462719 PMC10684634

[7] Meadows CIS Toolan M Slack A Newman S Ostermann M Camporota L. The Faculty of Intensive Care Medicine. Supplementary guidance for the diagnosis of death using neurological criteria when the patient is supported by extracorporeal membrane oxygenation In: Diagnosing death using neurological criteria. London: FICM (2021).

[8] Walte U Loewenbrück KF Dodel R Storch A Trenkwalder C Höglinger G. Recommendations of the German Society for Clinical Neurophysiology and Functional Imaging for the diagnosis of irreversible loss of brain function. Klinische Neurophys. (2019) 50:17–22. doi: 10.1055/a-2069-3379

[9] Bein T Muller T Citerio G. Determination of brain death under extracorporeal life support. Intensive Care Med. (2019) 45:364–6. doi: 10.1007/s00134-018-05510-z, PMID: 30627781

[10] Lie SA Hwang NC. Challenges of brain death and apnea testing in adult patients on extracorporeal membrane oxygenation-a review. J Cardiothorac Vasc Anesth. (2019) 33:2266–72. doi: 10.1053/j.jvca.2019.01.042, PMID: 30765209

[11] Sady ERR Junqueira L Veiga VC Rojas SSO. Apnea test for brain death diagnosis in adults on extracorporeal membrane oxygenation: a review. Rev Bras Ter Intensiva. (2020) 32:312–8. doi: 10.5935/0103-507x.20200048, PMID: 32667442 PMC7405745

[12] Thevathasan T Füreder L Donker DW Nix C Wurster TH Knie W. Case report: refractory cardiac arrest supported with veno-arterial-venous extracorporeal membrane oxygenation and left-ventricular Impella CP((R))-physiological insights and pitfalls of ECMELLA. Front Cardiovasc Med. (2022) 9:1045601. doi: 10.3389/fcvm.2022.1045601, PMID: 36407456 PMC9674118

[13] Migdady I Stephens RS Price C Geocadin RG Whitman G Cho SM. The use of apnea test and brain death determination in patients on extracorporeal membrane oxygenation: a systematic review. J Thorac Cardiovasc Surg. (2021) 162:e861:867–877.e1. doi: 10.1016/j.jtcvs.2020.03.038, PMID: 32312535

[14] Salih F Lambeck J Günther A Ferse C Hoffmann O Dimitriadis K. Brain death determination in patients with veno-arterial extracorporeal membrane oxygenation: a systematic study to address the harlequin syndrome. J Crit Care. (2024) 81:154545. doi: 10.1016/j.jcrc.2024.154545, PMID: 38395004

